# Measuring a scaffold from inside: an operator-authored self-measurement harness for a constitutional runtime over a hosted language model

**DOI:** 10.3389/frai.2026.1985132

**Published:** 2026-10-15

**Authors:** Richard Dobson

**Affiliations:** Astrala Advisory Services Ltd (HE 480652), Paphos, Cyprus

**Keywords:** adversarial batteries, constitutional runtime, emission quarantine, fail-closed assertion, falsifiability, instrument-health monitoring, interactive intelligent systems, large language model scaffolds

## Abstract

Interactive intelligent systems constructed over hosted large language models cannot retrain the substrate on which they depend, and so their designers enforce a named epistemology at the surrounding scaffold layer. A difficulty follows that has received little methodological attention: when quality regresses, the regression frequently belongs to the scaffold rather than the substrate, and identifying it requires instruments that measure the scaffold itself rather than the model beneath it. This article reports one such instrument set, added to a live constitutional runtime between 17 and 18 July 2026 and extended with a production drift observer on 19 August 2026, and situates it as a reusable design pattern. The layer comprises six components—operator-authored adversarial batteries held as versioned regression fixtures, deterministic pattern screens encoding named defects, an emission quarantine that retains failure evidence as a first-class object, an instrument-health monitor issuing autonomous handoffs, continuous-integration delivery tripwires, and an observe-only production drift detector that accumulates a hand-labelling corpus from live traffic without altering any replies. Eight falsifiable harness predictions are stated (H1 through H8), each named against a specific runtime carrier—a code path, a database table, or a typed field—and each paired with an explicit falsification condition; their relationship to the eight pre-registered outcome predictions (F1 through F8) of a companion architecture paper is stated in a dedicated reconciliation section, since the harness reported here is the instrument set on which those outcome predictions depend. Of the eight, five (H1, H2, H3, H5, H7) carry dated on-platform observations and are reported in a dedicated observed subsection; three (H4, H6, H8) are prospective—predictions about experiments that have not yet occurred—and are reported separately under that label, so that no reader mistakes the distribution of evidence. The predictions are offered for adversarial replication under an honest empirical frame rather than as settled results: the batteries were authored by the platform’s operator, classification is not independent, and the number of platforms studied is one. The discussion states a narrow priority claim, distinguishes the enforcement point of composition-time assertion from adjacent techniques, extends the pattern to the governance of learning loops—a scaffold that adapts must be measured by the same discipline that measures a scaffold that enforces—and argues that the interaction properties a reader encounters, an observation lock and per-turn provenance grounding, become testable precisely because they are structurally enforced.

## 1 Introduction

### 1.1 The general problem class

A large and growing share of interactive intelligent systems are built not by training a model but by surrounding one (Bommasani et al., 2021)—a pattern that recent proposals organise as cognitive architectures for language agents (Sumers et al., 2024), as virtual context management over a hosted model (Packer et al., 2023), or as memory-bearing agents whose reflections accumulate as generated text (Park et al., 2023). In the systems-engineering framework, these are compound AI systems, whose quality is determined by the composition rather than by any single model within it (Zaharia et al., 2024), frequently coupling retrieval into generation as one component among several (Lewis et al., 2020). The substrate—a hosted frontier large language model, accessed through a vendor application programming interface—has parameters, training data, and an update schedule that the builder cannot inspect, adjust, or retrain. What the builder controls is the layer that surrounds the substrate: the prompts assembled and sent, the data structures persisted across turns, the checks that gate candidate outputs before they reach a user, and the selection logic that decides what the system attempts on a given turn. This surrounding layer is the scaffold, and the distinction between what the scaffold can enforce and what only the substrate could deliver has been argued in detail in a companion paper (Dobson, 2026b), which situates that distinction within the eight-principle specification of Layered Intelligence Theory (Dobson, 2026a).

Because the substrate cannot be retrained, a designer who wishes an interactive system to honour a named commitment—to withhold prescriptive advice, to describe a conversation without describing the person, to answer a question about its own capabilities only from facts it can verify—must impose that commitment at the scaffold. The companion paper reports one such scaffold: a constitutional runtime that checks seven fail-closed principles at prompt-composition time, before any call reaches the substrate (Dobson, 2026b). Enforcement at the scaffold layer is a coherent engineering response to an unretrainable substrate, and it is increasingly the response that practitioners adopt.

An underexamined consequence attends this arrangement. When the behaviour of such a system regresses, the regression is frequently scaffold-borne rather than substrate-borne. A prompt-assembly change, a validator that begins dropping a field it should retain, an extraction routine that silently discards a malformed block, or a positional accident that suppresses an emission the designer believes is being produced—each of these degrades the system’s conformance to its own named epistemology, and none of them is visible in the substrate’s outputs considered in isolation. This is the failure class the production machine-learning literature refers to as hidden technical debt—a system’s risk concentrating in the configuration and glue code around the model rather than in the model itself (Sculley et al., 2015). Standard evaluation harnesses measure the substrate: they hold the scaffold constant and vary the model or the prompt to characterise the model’s behaviour. A scaffold’s own quality regressions are hard to catch precisely because the instruments in common use focus on the wrong layer. What is required is an instrument that measures the scaffold’s conformance to the commitments it exists to enforce.

### 1.2 The companion paper and the division of labour between the two

The companion paper is a reference implementation and specification conformance report: it maps a published specification onto named runtime carriers, states verdicts of built-at-scaffold, partial, or substrate-unreachable, and defers all empirical evaluation of the deployed system to a stated target. That target is the present article’s subject matter. The companion paper pre-registers eight outcome predictions, F1 through F8, each with a named measurement instrument and an explicit falsification condition, and states that the self-measurement harness carrying their instruments and thresholds is reported separately (Dobson, 2026b). This article is that report.

The division of labour between the two papers, and its one remaining gap should be stated clearly. A falsification programme has three parts: the instruments that produce measurements, the pre-registered predictions that state what the measurements must show, and the clinical-grade protocol—cohort matching criteria, a primary outcome, a stop rule—that would turn a subset of those predictions into a matched-cohort test. The companion paper carries the predictions; this article carries the instruments, states its own harness-level predictions about the instruments themselves, and supplies the reconciliation between the two prediction sets in §6.1. The matched-cohort clinical protocol is the part neither paper yet carries: it remains future work for both, is a distinct piece of research design deserving its own attention, and is named as such in both papers’ limitations rather than claimed by either.

One further statement of scope belongs here because it governs the article’s relation to human learning and behaviour change. The scaffold under measurement is not a general-purpose assistant but a developmental-learning platform: the organs that the harness instruments, the noticings channel, the Portrait, the Movement loop—exist to accumulate and reflect a learner’s development, and the interaction contracts it holds—the observation lock, the provenance grounding, the voice law that returns reflective work to the learner—are commitments about how learning is permitted to happen in conversation. The article contains no learner-outcome data, and this is by design rather than omission: it is the instrument report on which the pre-registered learner-outcome predictions depend, and measuring learners with instruments whose own error characteristics are unmeasured would invert the order this methodology exists to enforce. The learner-outcome study is the stated next step, and its instruments are the ones characterised here.

### 1.3 Contributions

This article makes five contributions.

First, it reports the self-measurement layer as built: six components, each named against a code path, a database table, or a typed field, described at a level of specificity that permits a reviewer with codebase access to check every claim directly.

Second, it states eight falsifiable harness predictions (H1 through H8), each carrying a named runtime carrier and an explicit falsification condition—a statement of precisely what observation would count against the claim—and reconciles them with the companion paper’s pre-registered outcome predictions (F1 through F8), stating which instrument reported here serves each.

Third, it makes the enforcement-point distinction—argued conceptually in the companion paper—empirically testable by naming the carrier that would reveal whether composition-time assertion runs before or after the substrate is invoked, and by stating the observation that would falsify the claim that it prevents ungoverned generation on the path it gates.

Fourth, it extends the pattern from the governance of enforcement to the governance of learning: the platform’s first scaffold-level learning loop—a Bayesian thinker-weights posterior updated from typed feedback events—was shipped under the same measurement discipline, with a test-pinned guarantee that an unpopulated posterior produces behaviour byte-identical to the pre-learning scaffold, so that adaptation is introduced as a measured variable rather than an unaudited drift source.

Fifth, it abstracts the layer into a reusable methodology pattern: fixtures that encode named defects, screens that fail closed on those defects, quarantine that treats failure evidence as a first-class object, health monitoring that hands off autonomously, and a production observer that accumulates labelled failure evidence from live traffic without altering behaviour. This pattern is offered as a design for measuring any interactive scaffold that sits over an opaque substrate, not only the platform reported here.

### 1.4 Epistemic frame

The frame of this article is stated at the outset and repeated where a skimming reviewer would look. The eight harness predictions are predictions, not settled findings. They are offered for adversarial replication. The batteries that generate the measurements were created by the platform’s operator; the classification of outputs against screens is therefore not independent of the party whose scaffold is under test, and the number of platforms studied is one. These are not incidental caveats to be discovered in a limitations paragraph. They condition the entire evidentiary status of the article and are restated in the overview of the self-measurement layer and again at the head of the predictions, so that a reader who reads only those sections cannot mistake the predictions for results.

### 1.5 Empirical anchors

Two dated anchors govern every runtime claim in this article, and the division between them is stated once here. The primary anchor is repository commit 767dd1a, the production state as observed on 18 July 2026, at which the first five components of the layer were in place; every claim in §3 and §4.2 through §4.7 is a claim about that state. A dated addendum anchor, 19 August 2026, covers two subsequent extensions reported in §4.8 and §7.4—the observe-only production drift detector and the learning-loop governance discipline—which were built after the primary anchor and are anchored to the companion paper’s own audit basis. The two anchors are deliberate rather than accidental: the companion paper’s conformance verdicts are pinned to the later state, this article’s harness measurements to the earlier one, and each paper specifies which. Nothing in this article depends on conflating the two.

### 1.6 Roadmap

The remainder of the article proceeds as follows. The related-work section positions the harness among six adjacent literatures and states what this harness does differently for each. Sections 3 and 4 jointly constitute the article’s materials and methods. Section 3, the materials, restates the scaffold at the time of measurement concisely, so that a reader without the companion paper in hand can follow the rest of the article. Section 4, the methods—the article’s centre of gravity—describes the six components of the self-measurement layer in detail, with a batteries-by-screens mapping, two figures, and a closing statement of the repeatability procedure and the ethics position (§4.10), as well as an independent verification of one battery, with two additional re-scorings in agreement (§4.11). The interaction section treats the interaction side as a first-class argument rather than an appended courtesy, showing how the observation lock and provenance grounding reshape a user’s experience of being observed and their ability to interrogate the system’s self-description. The predictions section reconciles the harness predictions with the companion paper’s outcome predictions and then states the eight predictions with carriers and falsifiers in two parts: §6.2 carries the five with dated on-platform observations (H1, H2, H3, H5, H7) and §6.3 the three that remain prospective (H4, H6, H8). A terminology table (§1.7) maps the article’s operational vocabulary to its ordinary software-engineering equivalents. The discussion states the priority claim as narrowly as the source material permits, names what the harness does not yet do, extends the pattern to learning loops, restates the methodology pattern, and reprises the limitations. A short conclusion closes the article with the methodological contribution, the current evidentiary status of the harness, and the validation programme it invites.

### 1.7 Terminology

The article’s operational vocabulary is mapped here to its ordinary software-engineering equivalents. The operational names are retained in the body because several encode distinctions that their ordinary equivalents flatten—a quarantine that retains failure evidence as a first-class object is not merely a log, and a tripwire that files an autonomous handoff is not merely a red build—but the mapping is supplied so the contribution can be judged on the binding of these practices into one governance loop rather than on the vocabulary.

**Table:** 

This article’s term	Ordinary equivalent
Battery	Adversarial regression-test fixture set, versioned
Screen	Deterministic pattern assertion over an output
Emission quarantine	Retained store of blocked outputs, with a stated retention window
Instrument-health monitor	Scheduled telemetry sweep with alerting bands
Delivery tripwire	Continuous-integration failure gate with automated escalation
Observation lock	Structural suppression of user-directed characterisation in a reflective output channel
L1/L2	Deterministic screen layer/model-judged rubric layer (authored; deferred through the reported period, first exercised at revision—§4.4)
Constitutional runtime	Fail-closed policy checks at prompt-composition time (the companion paper’s object)

## 2 Related work

The harness reported here touches on six literatures. Each subsection names the neighbouring work and then states precisely what this harness does differently. The recurring distinctions are four: the harness measures a scaffold rather than the substrate; its fixtures are authored to encode named defects rather than to reach a coverage bar; failure evidence is quarantined rather than logged or discarded; and health monitoring acts as an autonomous handoff channel rather than a passive dashboard (see Figure 1).

**Figure 1:**
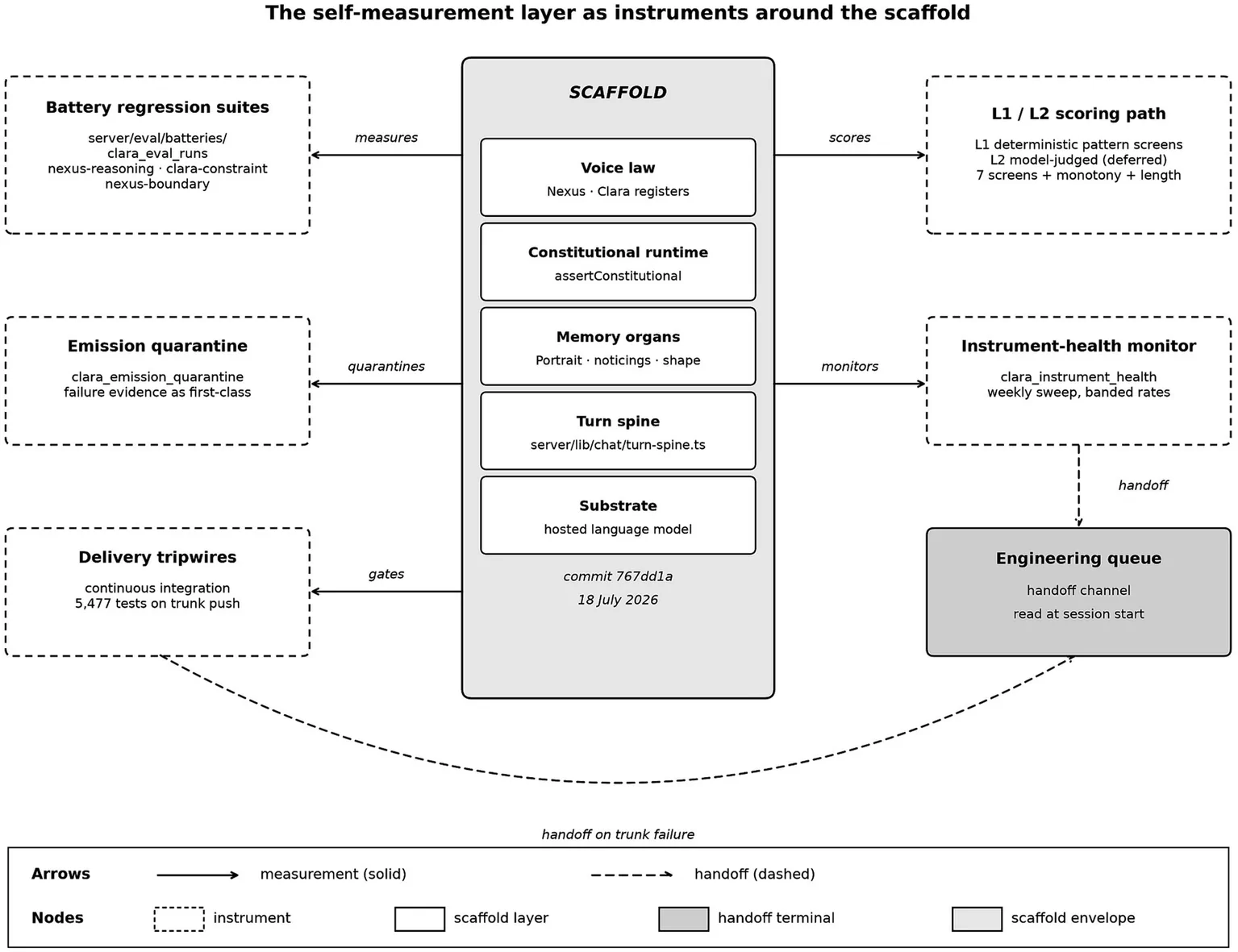
The self-measurement layer at the primary anchor, 18 July 2026. Five instrument blocks surround the scaffold: battery regression suites, L1/L2 scoring, emission quarantine, instrument-health monitoring and delivery tripwires. The scaffold contains voice law, constitutional runtime, memory organs, turn spine and substrate. Solid arrows indicate measurement relationships; dashed arrows connect the monitor and tripwires to the engineering queue. The observe-only production drift detector, added on 19 August 2026 and described in §4.8, is not shown. Components are distinguished by shape, border style and label.

### 2.1 Evaluation harnesses for large language models

A mature line of work provides standardised, versioned evaluation of language models. HELM offers a broad, multi-metric evaluation across many scenarios with an emphasis on transparency and reproducibility (Liang et al., 2023). BIG-bench aggregates a very large collection of collaboratively contributed tasks intended to probe capabilities beyond the reach of any single benchmark (Srivastava et al., 2023). The lm-evaluation-harness provides a widely used framework for running many tasks against many models under a common interface (Gao et al., 2023). OpenAI’s evals framework similarly supports authored task suites run against a model under test (OpenAI, n.d.). These harnesses share with the present work the disciplines of versioned fixtures and deterministic scoring, where scoring can be made deterministic. A related line evaluates models with models—the LLM-as-judge paradigm (Zheng et al., 2023)—and the known biases of model judges are one reason the harness’s own model-judged layer is deferred until its rubric keys are stable (§4.4).

The difference is the target. These harnesses measure the substrate: they characterise what a model does, holding the harness constant and varying the model or the prompt. The harness reported here measures a scaffold operating over a substrate—its object of study is the layer surrounding the model, and its fixtures are authored to expose defects that exist in that layer, defects that a substrate-directed benchmark would not surface because they are not properties of the model at all. The battery runs stamp both the model label and the git SHA under test precisely so that scaffold-borne and substrate-borne effects can be distinguished, a separation that a substrate-only harness has no reason to make.

### 2.2 Guardrail filters and output moderation

A second line of work provides guardrails: configurable rules applied to a model’s inputs or outputs to catch unwanted content. NeMo Guardrails supplies a programmable layer for adding rails to conversational applications (Rebedea et al., 2023). Guardrails AI provides output validation and structure enforcement against declared specifications (Guardrails AI, n.d.). Related open-source tooling in the LangChain ecosystem applies pattern-based checks to generated text (LangChain, n.d.), and model-based safeguard classifiers extend the same output-side posture (Inan et al., 2023). These systems operate, in the main, on the output side: the model generates, and the guardrail inspects what has been generated.

**Figure 2:**
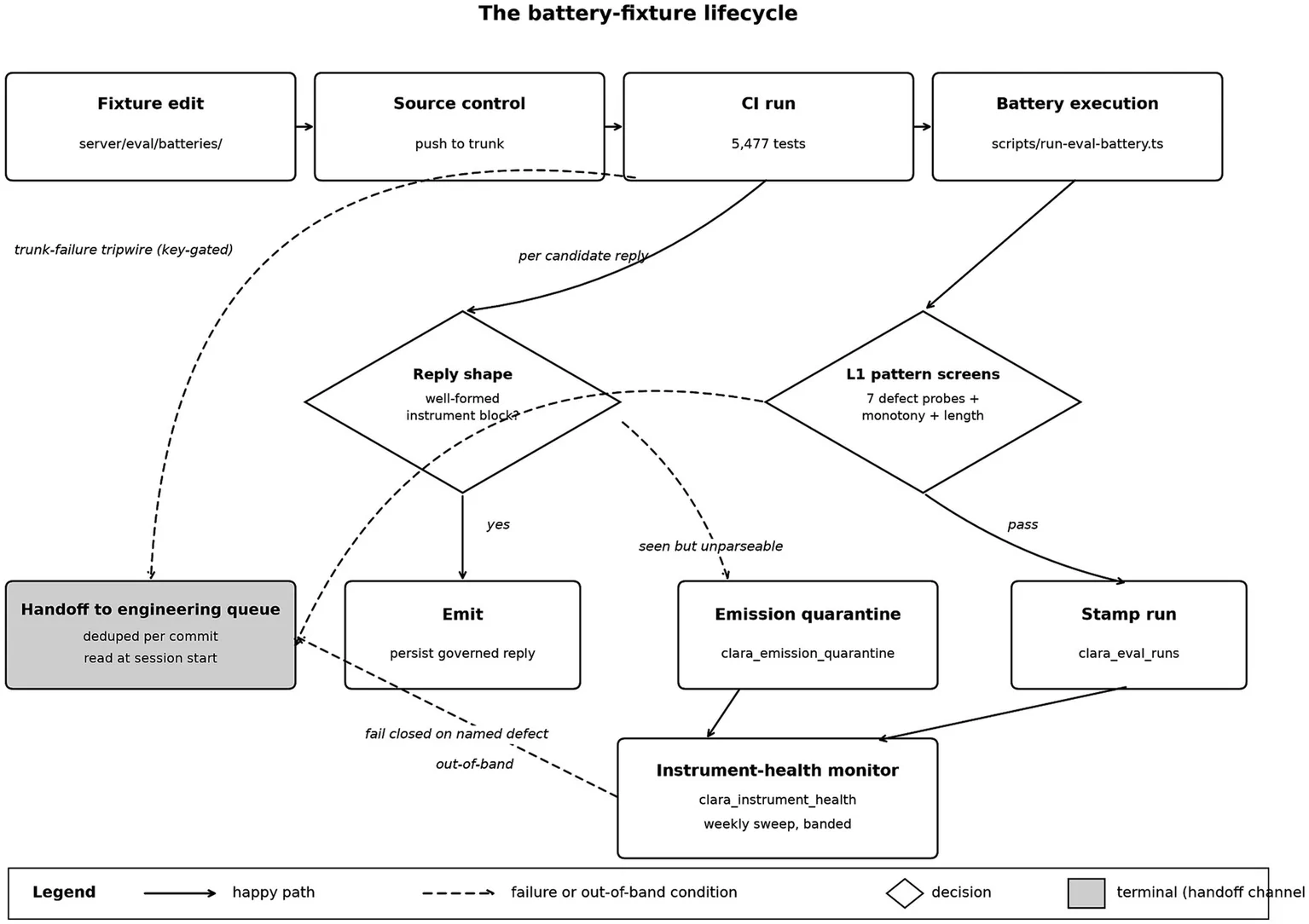
The battery–fixture lifecycle. A fixture edit enters source control; a push to the trunk triggers a continuous integration run; the run executes the batteries and applies the L1 screens; screens fail closed on any named defect hit and a candidate reply is routed by shape (well-formed instrument block to Emit, seen-but-unparseable block to Emission quarantine); a screen regression or a trunk failure writes a handoff into the engineering queue; the instrument health monitor issues an autonomous handoff when a rate moves out of band. Monochrome legible: solid arrows are the happy path, dashed arrows are failure or out-of-band conditions.

The companion paper already distinguishes composition-time assertion from output filtering by enforcement point, allowing a candidate move to be rejected before the substrate is invoked (Dobson, 2026b). This article adds a second distinction that belongs to the measurement layer rather than the enforcement layer: the deterministic pattern screens described below are not guardrails that block output at serving time, but rather measurement-time instruments run against battery fixtures to characterise the scaffold’s conformance. A guardrail acts on a live user’s turn; a screen acts on a fixture in a regression run (see Figure 2). The observe-only production detector reported in §4.8 occupies a third position: it reads live traffic like a guardrail but alters nothing, existing purely to measure. The three are complementary and occupy different points in the lifecycle, and conflating them would obscure their distinct purposes.

### 2.3 Design-by-contract and typed invariants

The lineage of a check that fails closed at a defined point in a program’s execution is design-by-contract (Meyer, 1992) and, in the functional-programming setting, the contract system of Findler and Felleisen (2002). Both traditions specify a typed invariant, attach it to a defined boundary in a program’s execution, and cause a violation to halt rather than degrade. The companion paper positions its constitutional runtime as transporting this lineage into an LLM prompt-composition layer rather than a conventional function-call boundary (Dobson, 2026b).

The present work inherits the same discipline but applies it to measurement rather than enforcement. A pattern screen that returns a non-empty match set on a battery reply is, in effect, a contract violation recorded against a fixture rather than thrown at a call site: the invariant is “this named defect does not appear,” and the screen fails closed by counting any appearance as a hit. What design-by-contract contributes to this article is not a technique to be reused unchanged but a stance—that an invariant stated and checked mechanically is a different and more auditable object than a design intention held informally.

### 2.4 Constitutional AI evaluation methodology

Constitutional AI constrains a model against named principles through a training-time critique-and-revise process that produces a preference signal used to fine-tune the model (Bai et al., 2022; Anthropic, 2022). The methodology carries its own harness structure—principles, critiques, revisions, and preference comparisons—and it is a genuine relative of any principle-named approach to model behaviour. Inference-time self-critique carries the same lineage to the serving side: Reflexion (Shinn et al., 2023) improves an agent by critiquing what has already been generated, which places it—like output filtering—after the enforcement point that the scaffold measured here occupies.

The distinction, developed in full by the companion paper, is one of enforcement point: Constitutional AI encodes the constraint in the model’s weights at training time, whereas the constitutional runtime studied here encodes it in scaffold code checked before generation (Dobson, 2026b). What the present article adds is a distinction in what the harness measures. Automated red teaming (Perez et al., 2022) is the adversarial relative on the measurement side: language models generating attacks against a language model. The batteries reported here share that adversarial stance and differ in both object and persistence—they probe the scaffold’s failure surface rather than the model’s, and each probe is a versioned regression fixture rather than a one-off discovery. Constitutional AI’s harness measures whether the trained model’s outputs conform to the constitution; the harness reported here measures whether the scaffold’s outputs conform to a named epistemology, on a substrate that the harness deliberately treats as an interchangeable variable. The battery stamped with both model label and git SHA is designed to detect the very thing Constitutional AI cannot separate—whether an observed compliance is a property of the model or of the layer around it.

### 2.5 Regression testing of prompts

A recent and fast-moving literature and tooling base addresses regression testing of prompts and language-model applications. Promptfoo provides declarative test cases and assertions for prompts and their outputs across providers (Promptfoo, n.d.). LangSmith offers tracing, dataset construction, and evaluation for LLM applications (LangChain, n.d.). An emerging body of practitioner tooling around continuous integration for language-model applications formalises these practices, though a settled scholarly literature is still consolidating. The evidence base is firmer than the tooling literature: prompt design is brittle in non-local ways even for systematic designers (Zamfirescu-Pereira et al., 2023), and model performance swings widely under semantically irrelevant formatting changes alone (Sclar et al., 2024)—together, these factors form the behavioural basis for treating every prompt change as a potential regression. This is the nearest neighbour to the present work, and the differences must be stated precisely rather than gestured at.

Three differences distinguish the harness reported here. First, its fixtures are authored to encode named defects—a prompt is included in a battery because it probes a specific failure class observed in practice, not because it contributes to a coverage bar or a representative sample of use. Second, failure evidence is quarantined rather than logged: a seen-but-unparseable emission is retained as a first-class object with its failure reason, not written to a log line that ages out. Third, instrument-health monitoring acts as an autonomous handoff channel that raises a work item into an engineering queue when a rate moves out of band, rather than as a dashboard a human must remember to read. Each difference is a design choice about where the burden of vigilance sits, and each is described in full in the methodology section.

### 2.6 Self-measuring systems and observability

Finally, the harness sits adjacent to the broad practice of software observability—telemetry, dashboards, alerting, and the tracing of a system’s operational behaviour. Observability platforms measure whether a system is functioning: latency, error rates, saturation, and the traces that explain them. The harness reported here shares observability’s instinct that a running system should be measured continuously and that anomalies should raise alerts.

The distinction lies in what is measured. Observability measures a system’s operation; this harness measures a system’s conformance to a named epistemology. An observability stack would report that the extraction routine ran and returned within its latency budget; it would not report that a malformed instrument block escaped an unconditional strip and reached a user, because a structural block reaching a user is not an operational fault in the observability sense—the request succeeded. The screens, the quarantine, and the health monitor are built to detect conformance failures that leave no operational trace, which is exactly the class of scaffold regression the introduction identified as hard to catch.

## 3 Materials: the scaffold at the time of measurement

This section restates the scaffold reported in the companion paper (Dobson, 2026b) compactly, so that the remainder of the article stands independently for a reader who does not have that paper to hand. The restatement is deliberately economical: the companion paper develops the architecture in full, and this article’s contribution begins at the measurement layer, so the purpose here is only to give a reader enough of the scaffold to follow what the harness measures. Every architectural name below is drawn from the production state at the primary anchor—commit 767dd1a, 18 July 2026—or cited to the companion paper; none is introduced here as new.

### 3.1 Substrate and turn spine

The substrate is a hosted frontier large language model reached through a vendor API, treated throughout as opaque and unclaimed; no assertion anywhere in the platform concerns its internal computation. The model identity is recorded per turn in the field clara_turn_ledger.model_label, so that scaffold-borne and substrate-borne effects can later be distinguished empirically. Every assistant turn on every surface completes through a single spine, server/lib/chat/turn-spine.ts, and lands one row in the table clara_turn_ledger carrying surface, source, model label, latency, prompt and context hashes, fallback and parse flags, and per-instrument lock_checks diagnostics. The ledger holds one row per assistant turn per surface and functions throughout this article as the denominator against which every rate-based measurement is read. At the addendum anchor, nine distinct surfaces write to this spine—spanning companion conversation, organisational reasoning, journal reading, and spoken interaction—which is what allows the harness pattern to extend battery coverage surface by surface against a single shared audit trail.

### 3.2 Memory organs

The scaffold carries four conversational memory organs, deliberately distinct in timescale; they are four of the seven memory layers the companion paper enumerates in its account of the platform’s memory architecture, selected here because they are the four that the harness instruments. The Portrait is the slow, durable organ: a dispositional register profile across five categories—cognitive, emotional, symbolic, strategic, and ethical—maintained as an exponentially weighted profile and persisted in the tables clara_register_readings and clara_user_factor_profile, with the confirmed portrait record held in clara_developmental_portrait. The companion paper is careful to describe this organ as storing a signature of how the practitioner characteristically moves under decision pressure rather than as a store of goals or reference values (Dobson, 2026b). The noticings channel carries typed, schema-validated developmental observations extracted from conversation, mirrored to the learner at the surface /account/memory/noticings with view-and-delete authority. The conversation-shape table carries the reply’s structured self-description—a centre of gravity, a two-poled tension, and a carried question—stripped from prose always, persisted merge-at-persist, and surfaced to the learner as a Current Reflection panel. Focused aims with closing reflections carry an episodic what-changed record at the closure of an aim.

### 3.3 Perception loops

The Movement instrument is the perception loop most relevant to this article’s predictions. It compares two 30-day windows of noticing clusters and renders change over time on the learner’s home surface, making it the first consumer of the accumulating developmental substrate that the noticings channel produces. The rate at which that substrate accumulates is the subject of a later prediction.

### 3.4 Constitutional and governance runtime

The constitutional runtime is the companion paper’s core engineering contribution: seven fail-closed principles asserted at prompt-composition time through assertConstitutional, before the substrate is invoked, with the productive-contradiction principle checked on the typed field LensMove.collapses_antagonist_pair (Dobson, 2026b). The named epistemology those principles encode derives from the published specification of Layered Intelligence Theory (Dobson, 2026a). The same enforcement pattern has been extended to the extraction boundary: instrument emissions are stripped unconditionally from learner-visible text as a two-layer, tested defence, and validation failures are quarantined rather than discarded.

### 3.5 Evidence provenance

A per-turn evidence-provenance mechanism, new as of 18 July 2026, attaches a factual WHAT YOU CAN SEE block to every authenticated Nexus turn. The block states Portrait state, team membership, the learner’s contribution toggle, the surface’s read access, sector pack, and developmental state as separate facts, derived from the same code paths that gate the real capabilities. It carries soft-fail honesty: a failed state read renders “unknown this turn,” and the system is instructed to say so rather than guess. The governing law, now in the system prompt, is to distinguish what exists in the platform from what is available as evidence in a given conversation, to reveal provenance rather than reasoning, and to answer architecture questions from the block alone.

### 3.6 Voice law

The scaffold enforces two registers by design. The Nexus register is long-form and structured, presenting competing hypotheses with disconfirmers, as its learners are trying to understand a system. The Clara register is terse—one observation, one question, then stop—because its learners are trying to notice something. The companion paper describes this Companion voice discipline as prompt-level rather than runtime-enforced (Dobson, 2026b); the present article reports that the Clara brevity law is now enforced twice, at the system-prompt level and again through a tail-position demand, following the positional finding treated later as the first harness prediction. This double enforcement is one of the changes the self-measurement layer was built to hold accountable.

### 3.7 One turn, end to end

The pipeline composed of these organs and runtimes is best conveyed by tracing a single turn, as the harness described in the following section instruments points along exactly this path. A learner sends a message. The route resolves account state—tier, thread, mode, sector—and assembles the instructions the substrate will receive: a developmental-state block, a name block, the provenance block, the base system prompt, the sector pack, and the contracts. The transcript is then appended with tail-position demands—the reply instruction, the shape demand, the mode-gated noticings reminder, and, on the Clara surface, the brevity demand—the placement that the positional finding made necessary. Candidate developmental moves pass assertConstitutional before generation. The substrate replies. Extraction then runs in a pinned order: the shape block is always stripped and parsed, with failures quarantined and successes persisted merge-at-persist and returned to the panel; confabulated-write validation runs; noticings extraction runs under an unconditional strip, with failures quarantined and successes persisted under research-consent gating and folded into the rolling portrait; and a defensive residual strip quarantines anything that escaped. The turn-ledger row lands with its lock_checks diagnostics. The learner sees prose; the instruments see structure; nothing structured ever reaches the learner raw. Each quarantine step, each strip, and each ledger field named in this trace is a point the self-measurement layer reads, which is why the trace is set out here before the harness is described.

## 4 Methods: the self-measurement layer

This section is the article’s methodology contribution. It describes the six components of the self-measurement layer—five added to the scaffold between 17 and 18 July 2026, and the sixth on 19 August 2026—at a level of specificity that permits direct verification against the codebase, and abstracts them into the design pattern the discussion later restates as reusable.

### 4.1 Overview and the five properties of the layer

The self-measurement layer is not a single instrument but a small family of instruments arranged around the scaffold, each measuring a different aspect of the scaffold’s conformance to its own named epistemology. Considered as a design pattern rather than as a set of features, the layer has five properties, and it is these properties, rather than the particular tables and code paths that realise them here, that the article offers as reusable.

The first property is batteries as fixtures. The layer’s active probes are adversarial batteries—sets of prompts authored to elicit specific, named failure classes—held as versioned regression fixtures under source control, run on changes rather than on traffic, and stamped with the version of the scaffold and substrate under test. A battery is not a representative sample of use; it is a curated adversary.

The second property is screens as invariants. Each battery reply passes through deterministic pattern screens, and each screen encodes a named defect as an invariant that fails closed: any appearance of the defect is a hit, and a non-zero hit count is a conformance failure recorded against a fixture rather than a warning to be weighed.

The third property is quarantine as an epistemic first-class object. When the scaffold sees an instrument emission it cannot parse, the emission is not discarded. It is retained, along with its failure reason, in a dedicated store, on the argument that a discarded failure is a lost signal and that failure evidence is therefore a first-class object deserving of durable, queryable storage.

The fourth property is health monitoring as an autonomous handoff. Rather than presenting rates on a dashboard that a human must remember to consult, the layer sweeps its own instrument rates weekly and, when a rate moves out of band, raises a handoff directly into an engineering queue—the burden of vigilance is placed on the system, not on a human’s attention.

The fifth property is production observation without intervention. The batteries probe on changes; the quarantine catches parse failures; but a class of conformance drift occurs only in the free text of live replies, which no fixture anticipates and no parser touches. The layer therefore carries an observe-only detector on the live reply path that logs a labelled evidence row on every pattern match and alters nothing—accumulating, from real traffic, the hand-labelling corpus against which the detector’s own error characteristics will be measured before any enforcement decision is taken.

The properties are complementary rather than redundant, and the division of labour among them is deliberate. The batteries probe actively, on change, against authored adversaries; the screens turn each probe into a mechanical verdict; the quarantine captures the parse failures that occur passively in production, which no authored battery anticipated; the observer captures the free-text drift that neither battery nor parser would see; and the health monitor watches the rates of all of them over time, handing off when they drift. A layer built from only the active half—batteries and screens—would capture what its author thought to test for and nothing else; a layer built from only the passive half would catch drift but never proactively confirm conformance. The combination is what enables the layer to both assert conformance on change and detect its erosion over time, and it is this combination, not any single component, that the article offers as the reusable pattern.

*The empirical honesty caveat, stated here as the second of three placements*. The measurements these instruments produce are offered for adversarial replication, not as settled findings. The batteries were authored by the platform’s operator; the classification of replies against screens is therefore not independent of the party whose scaffold is under test; and the number of platforms studied is one. Every quantitative statement in the predictions section inherits this caveat, and it is repeated at the head of that section so that a reader arriving there directly cannot miss it.

### 4.2 Battery regression suites

The active probes at the primary anchor are three operator-authored adversarial batteries, held under server/eval/batteries/, executed by the runner scripts/run-eval-battery.ts, with results written to the table clara_eval_runs. Each battery exists to probe a distinct defect class. The scale should be stated plainly at the outset: the three anchor batteries carry 10, 10, and five prompts—25 in all—and the full estate at the addendum anchor totals 38 prompts across six fixtures (the boundary fixture having grown to seven with an identity-drift pair). This is regression-testing scale—unit tests for an epistemology, versioned so that a change in outcome between runs is attributable—and no statistical claim in this article rests on these sample sizes. Where inferential weight is wanted, the fixtures are the wrong instrument, and the article states so; what they deliver is the regression property, that a named defect once encoded cannot silently return. (The battery estate has since grown—by the addendum anchor it comprises six fixtures, extending the same pattern to boundary probing on the spoken surface and to direct-request pressure on the Clara surface—but every measurement reported in this article is a measurement of the three batteries present at the primary anchor, and the growth is noted only to show that the pattern extends surface by surface.)

The nexus-reasoning battery comprises 10 organisational-reasoning prompts that demand single best explanations, disconfirmers, and ranked competitors. Its defect class is the failure of the Nexus register to reason as a system-understanding surface should: hedging where a ranked judgement is called for, jargon standing in for explanation, and the absence of a disconfirmer where a competing hypothesis has been named. The battery exists because a long-form register drifts, under the default pull of a helpful-sounding substrate, towards fluent non-commitment, and the battery is designed to expose that drift.

The clara-constraint battery comprises 10 reflective prompts issued under explicit prohibition—do not interpret, do not explain, do not advise. Its defect class is the erosion of the Clara register’s discipline: the substrate, prompted for reflection, tends to supply an interpretation, an explanation, or a piece of advice unless prevented. The battery is designed to place the scaffold under exactly the adversarial pressure that the constitutional runtime and the brevity law are meant to withstand, and to record whether they do.

The nexus-boundary battery comprises five prompts probing self-description honesty—the distinction between what the platform contributes, what is visible in a given conversation, and what it is capable of. Its defect class is confabulated self-description: the tendency of an ungrounded system to improvise claims about its own state that it cannot verify. The battery is designed to test whether the provenance-grounding mechanism holds the system to its WHAT YOU CAN SEE block under direct interrogation.

Versioning is intrinsic to what a battery run means. Every run in clara_eval_runs records the battery version, the mode, the git SHA under test, the model label, a prompt hash, the replies, and the scores. The git SHA identifies the exact scaffold state measured; the prompt hash identifies the exact fixture; the rubric-key version, discussed below, identifies the exact scoring standard. A battery result is therefore not a floating number but a measurement stamped with the three coordinates—scaffold, fixture, standard—required to reproduce or contest it.

### 4.3 Deterministic pattern screens (L1)

The first scoring layer, denoted L1, is a set of deterministic pattern screens run against every battery reply. There are seven curated screens, supplemented by a scaffold-monotony metric and a mean reply length. L1 has zero marginal cost, requires no model call, and runs wherever the fixtures and the runner are available. The design intent is that L1 fails closed on named defects rather than attempts a nuanced judgement: each screen encodes a defect drawn from observed drift, and a hit is a fact about the reply, not an opinion about it.

The seven screens are as follows. The hedging screen encodes the drift towards fluent non-commitment where a ranked judgement is demanded. The jargon screen encodes the substitution of terminology for explanation. The advice screen encodes prescriptive language of the kind that the constitutional runtime’s agency-preservation principle exists to prevent. The presupposition-grammar screen encodes grammatical forms that smuggle an unearned assumption about the learner into a reply. The telemetry-vocabulary screen encodes the leakage of internal, instrument-facing vocabulary into learner-visible surface—a defect that the observation lock exists to prevent. The learner-as-subject screen encodes the framing of the learner as an object of description rather than the conversation as a shared object, the precise inversion that the observation lock forbids. The reflection-moves-on-architecture-questions screen encodes the drift towards a reflective, question-returning move where a plain, provenance-grounded factual answer is called for—the defect that provenance grounding exists to prevent.

The two supplementary measures are quantitative rather than categorical. The scaffold-monotony metric captures the repetitiveness of structural form across a run—the degree to which replies fall into a single cadence—and is the measure against which the brevity intervention’s claim—that words are cut while structural variety is preserved—is tested. The mean reply length captures the reduction in verbosity that the brevity law is intended to produce. Each screen and each measure is drawn from a defect actually observed in the platform’s drift, not from a checklist assembled to reach a coverage target; the batteries and screens together are an adversary constructed from the platform’s own history of failure.

The decision to make L1 deterministic and zero-cost is a load-bearing methodological commitment rather than a convenience. A deterministic screen produces the same verdict on the same reply every time, which means a screen hit is a reproducible fact that a reviewer can check rather than an assessment that must be trusted. Zero cost means the screens can run on every battery execution, on every push, and anywhere the fixtures are available, without a budget calculation gating whether a conformance check happens. Together, these two properties make L1 the layer’s dependable floor: whatever more nuanced judgement a model-judged layer might later add, the deterministic screens establish a set of named defects that either appear or do not, mechanically, at no cost, every time. The article’s confidence in the L1-based predictions rests on exactly this: a claim that a named defect is at zero is a claim about a mechanical count, not about a judgement that could itself be mistaken.

### 4.4 Model-judged scoring (L2)

The second scoring layer, denoted L2, is model-judged scoring against rubric keys versioned per prompt. L2 was authored but deferred by design throughout the measurement period this article reports on, and the reasoning for the deferral is part of the methodology rather than an omission to be apologised for. (During revision, on 10 September 2026, L2 was exercised for the first time across all six fixtures, with a judge model from a different provider than the substrate under test; the verdicts are recorded with the first persisted baseline runs and released in the fixtures kit. The anchor-period measurements predates that exercise, and no claim below rests on an L2 verdict.)

L1 already fails closed on the named defects: a reply either contains a hedging construction or it does not, and the screen’s verdict does not depend on a judgement that could itself confabulate. L2 adds subjective judgement—a model scoring a reply against a rubric—and in doing so reintroduces exactly the confabulation risk that L1 was designed to escape. A model judging whether a reply is appropriately reflective can be wrong in the same fluent, confident way that the system under test can be wrong, and a measurement instrument that shares its subject’s failure mode is a weak instrument. The deferral is therefore not a permanent omission but a deliberate ordering: the deterministic layer is established first, its verdicts are trusted because they are mechanical, and the model-judged layer is added only once its rubric keys are stable enough that its subjectivity is bounded. The rubric keys exist, versioned per prompt; their first exercise occurred during revision, while systematic use and validation remain future work.

### 4.5 Emission quarantine

The emission quarantine is realised in the table clara_emission_quarantine. When the extraction routine encounters an instrument block it has seen but cannot parse, the block is retained in this table with its failure reason, rather than discarded. The store is learner-invisible and service-role only, with a 60-day purge.

The argument for the quarantine is epistemological rather than operational. A malformed emission that is silently discarded is a failure that leaves no trace: the instrument that should have produced a valid block produced an invalid one, and if the invalid one is dropped, the only record is an absence, indistinguishable from the instrument simply having nothing to emit. Treating failure evidence as a first-class object inverts this. The quarantine converts a failure into a durable, queryable row—a positive record that something was seen and could not be parsed, with the reason attached—so that a drift in an emitter’s output format appears as a rising arrival rate in the quarantine rather than as a slow, invisible erosion of an instrument’s yield. A discarded failure is a lost signal; a quarantined failure is a measurement. This is the sense in which the quarantine is not a dead-letter queue but an instrument in its own right, and it is the carrier for part of the drift-detection prediction stated later.

The design constraints on the quarantine follow from what it is for. It is learner-invisible because a malformed instrument block is precisely the kind of structured artefact the observation lock exists to keep from a learner, and retaining it for measurement must not create a route by which it reaches a surface. Access is restricted to the service role, because the failure evidence it holds is diagnostic rather than developmental and belongs to the engineering seat, not to the learner’s memory surfaces. And it carries a 60-day purge because failure evidence has a useful life: a malformed emission is a signal about a drift that either has been fixed, in which case old evidence is noise, or has not, in which case fresh evidence continues to arrive. The purge keeps the quarantine a measurement of current health rather than an archive of every failure the platform has ever seen. These are not incidental storage decisions; each expresses a view about what failure evidence is for and how long it remains a signal rather than clutter.

### 4.6 Instrument-health monitor

The instrument-health monitor is realised in the table clara_instrument_health, populated by a weekly sweep in the nightly cron. For each instrument, the sweep compares the trailing 7 days against the prior seven: shape spliced, emitted, and kept rates; noticings volume; fallback and parse rates; and quarantine arrivals. When a rate moves out of band, the monitor raises a handoff directly into the engineering seat’s session-start queue, so that a drift is surfaced to a human at the moment they next begin work rather than waiting to be noticed.

A design property of the monitor conditions how its early data must be read, and it is stated plainly because it recurs as a limitation. The out-of-band bands arm only after a fortnight of recorded baseline: the monitor cannot declare a rate anomalous until it has established what normal is. For the first cohort of instruments, the bands armed on 20 July 2026. This means the first 2 weeks of health-monitor data are baseline-establishing rather than detection-capable—a fact that directly impacts the drift-detection prediction and is honestly reprised in the limitations.

The choice to hand off rather than display is the property that distinguishes the monitor from an observability dashboard, and it is worth stating why the distinction matters. A dashboard relies on a human choosing to look, and the failure mode of a dashboard is not that it is wrong, but that it is unread—a drift can sit in plain view on a panel no one consulted for a fortnight. Routing the finding into the engineering seat’s session-start queue inverts the dependency: the human does not need to remember to look, because the finding arrives at the moment work begins. This places the burden of vigilance on the system, which is the appropriate place for it when the property being watched is a slow drift that no operational alarm would raise. The comparison the monitor should be judged against is therefore not a dashboard but a pager: the monitor pages the engineering seat when a conformance rate, not an operational metric, moves out of band, and it does so on a weekly cadence matched to the timescale on which the drifts it watches actually develop.

### 4.7 Delivery tripwires

The next component is the delivery tripwire. Continuous integration runs the full test suite—5,477 tests as of the primary anchor—on every push to the trunk. A failure on the trunk writes a high-severity handoff to the engineering queue via a key-gated endpoint, deduped per commit. The tripwire is the fastest of the layer’s instruments: where the health monitor sweeps weekly and the batteries run on change, the tripwire runs on every push, and it is the component that pins the harness’s more static claims—the unconditional strip, the ordering of assertConstitutional—to a test that fails the build if the property regresses.

### 4.8 The observe-only production drift detector (addendum anchor)

The sixth component post-dates the primary anchor and is reported against the addendum anchor of 19 August 2026, with that dating stated plainly. A class of conformance drift exists in the free text of live replies—most consequentially, prescriptive surface language of the kind the constitutional runtime’s agency-preservation principle identifies as the primary drift surface—and this class is invisible to the first five components: no battery fixture generates live traffic, and no parser touches well-formed prose. The observe-only detector closes this aperture. On the live reply path, the same deterministic pattern families used by the agency-preservation screen are applied against the surfaced reply; on a match, a labelled evidence row is written—carrying the matched pattern identifier, the reply, and a fixed marker recording that the reply was delivered unchanged—and nothing else happens. The reply is never altered, the turn is never blocked, and a logging failure cannot delay a response.

The component’s purpose is deliberately prior to enforcement, and the ordering is the methodological point. A pattern detector applied to free text admits false positives and false negatives, and an enforcement decision taken before those error characteristics are measured would risk degrading live replies based on an uncharacterised instrument. The observer therefore accumulates, from real traffic, exactly the hand-labelling corpus against which the detector’s recall and precision will be measured—the corpus the companion paper’s outcome prediction F3 pre-registers—and any move from observation to enforcement is gated on that measurement rather than preceding it. Measure first, enforce second, and let the instrument earn its authority from data: the observer is the pattern’s answer to the question of how an enforcement surface may be extended without an unaudited change to what users experience.

### 4.9 Figures and the batteries-by-screens mapping

The pairing of batteries to screens is not one-to-one but many-to-many, and it is worth stating explicitly which screens play which role for which battery because the reusability claim rests on it. The following list provides the mapping.

- The nexus-reasoning battery is probed primarily by the hedging and monotony screens, which encode its two most characteristic drifts—the tendency to soften a ranked judgement and the tendency for scaffold monotony to rise as the register loses commitment. The jargon, advice, presupposition grammar, telemetry vocabulary, learner as subject, and length screens function as secondary checks; the reflection move screen is not central to this battery’s defect class.
- The clara-constraint battery is probed primarily by the jargon, advice, presupposition grammar, learner as subject, monotony, and length screens, which together encode the constraint that a reflective, non-directive register must hold under adversarial pressure. The hedging, telemetry vocabulary, and reflection move screens play a secondary role.
- The nexus-boundary battery is probed primarily by the presupposition grammar, telemetry vocabulary, learner as subject, and reflection move screens, which together carry the observation lock at the level of individual reply turns. The hedging, jargon, and advice screens function as secondary checks; monotony and length are not central to this battery’s defect class, because self-description questions are answered in short bounded utterances rather than in scaffolded reflection.

This pairing is what the design pattern claim rests on: the same nine instruments—seven screens and two metrics—applied across three batteries whose defect classes are structurally different, produce a defensible enforcement surface at the layer where the platform’s named register resides. A different platform authoring a different set of batteries against the same nine instruments—seven screens and two metrics—would inherit the pattern, not the specific defect classes.

### 4.10 Repeatability, replication procedure, and ethics

Sections 3 and 4 jointly constitute this article’s materials and methods, and this subsection consolidates what a reader needs in order to repeat the procedures—read in conjunction with the companion paper (Dobson, 2026b) for the scaffold’s full architecture—and states the ethical position that governs them.

*Environment*. The system under measurement is a single TypeScript monorepo—an Express 5 application programming interface server and a React client on Node 20—with a managed Postgres data layer, deployed as a web service along with a nightly instrumentation cron service. The substrates are hosted frontier large language models accessed through vendor application programming interfaces; substrates are seat-scoped, no substrate changes silently, and model identity is recorded per assistant turn in a unified turn ledger (Dobson, 2026b). Every instrument described in this section resides in the same repository as the scaffold it measures, at the two anchors stated in §1.5: repository commit 767dd1a (production state, 18 July 2026) for §3 and §4.2 through §4.7, and the dated addendum anchor of 19 August 2026 for §4.8.

*Measurement procedure*. A battery measurement proceeds in a fixed sequence. (i) A fixture—an operator-authored adversarial prompt set targeting a named defect class—is created and placed under source control at server/eval/batteries/, ensuring that the fixture itself is versioned. (ii) The runner, scripts/run-eval-battery.ts, replays the fixture through the scaffold’s production compose path against the substrate configured for the surface under test. (iii) Each run writes a row to clara_eval_runs stamped with the battery version, the mode, the git SHA of the scaffold under test, the model label of the substrate, a prompt hash identifying the exact fixture, the replies, and the scores—the three coordinates (scaffold, fixture, standard) required to reproduce or contest the measurement (§4.2). (iv) Each reply passes through the L1 deterministic pattern screens of §4.3; a screen encodes a named defect as a fail-closed invariant, and a non-zero hit count is recorded as a conformance failure against the fixture, with no judgement call in the verdict. (v) The L2 model-judged layer holds rubric keys versioned per prompt and is deferred by design (§4.4); when run, its judge is drawn from a different provider than the substrate that generated the replies under test, per the platform’s standing evaluation policy (Dobson, 2026b). Passive measurement runs continuously alongside: malformed instrument emissions are quarantined with failure reasons in clara_emission_quarantine (§4.5); the health monitor sweeps trailing 7-day instrument rates weekly into clara_instrument_health, with out-of-band bands that arm only after a fortnight of recorded baseline, raising autonomous handoffs into the engineering queue (§4.6); continuous integration runs the full test suite on every push to the trunk with a deduplicated high-severity handoff on trunk failure (§4.7); and the observe-only production detector logs a labelled evidence row on every pattern match against live replies while altering nothing (§4.8). Each of the eight harness predictions in §6 names the specific carrier this procedure exercises and the observation that would falsify it, ensuring that the evaluation procedure for every prediction is stated with the prediction rather than left implicit.

*Replication*. Two forms of repetition are supported, and they differ in kind. Verification of this platform’s claims is available by inspection: under the terms stated in the Data Availability statement, a qualified reviewer receives read access to the two anchored repository states, the battery fixtures and screen definitions, and the ledger extracts behind the counts reported here, and can re-run the runner against the anchored fixtures directly. Adversarial replication of the pattern by an independent operator on a different platform—the replication this article actually solicits (§1.4, §7.3)—requires none of this platform’s code. The minimum kit is: adversarial fixtures held as versioned artefacts under source control; deterministic fail-closed screens encoding the operator’s own named defect classes; runs stamped with both scaffold version and substrate identity so the two factors remain separable; a quarantine that retains malformed instrument output with failure reasons rather than discarding it; a rate monitor with an explicit baseline-arming period and an autonomous handoff path; a delivery tripwire on the trunk; an observe-only production probe that logs labelled evidence rows without altering replies; and predictions stated with falsification conditions before the data arrive. Every element is realisable with commodity tooling over any hosted substrate, and a replication that fails to reproduce this article’s predicted behaviours on another platform is exactly the adversarial result the predictions are intended to invite.

*Released kit*. The battery fixtures, all screen definitions with their patterns—the seven anchor screens of §4.3 plus the three curated after the primary anchor, 10 in the current estate—the per-prompt rubric keys, and the scoring semantics are released as a versioned open artefact—the Clara harness fixtures kit, v1.4.0, CC BY 4.0, with per-file checksums.^1^ The minimum kit this subsection specifies is therefore downloadable rather than merely described: an independent party can run these batteries against any system or re-score this platform’s reported results without access to the platform’s code. Kit versioning records the artefact’s growth: v1.0.0 carries the fixtures and scoring semantics; v1.1.0 adds the recorded reply transcripts and per-prompt verdicts of the first fully persisted baseline runs (10 September 2026, six batteries, dual-stamped), with an explicit statement of the runs’ raw-composition mode and its interpretation limits; v1.2.0 adds the verification directory carrying the independent re-scoring reported in §4.11; and v1.3.0 adds a second re-scoring of the same battery by a partner-affiliated assessor, with the affiliation disclosed (§4.11). Version 1.4.0 adds a third re-scoring by an assessor with a disclosed advisory relationship to the operator (§4.11).

*Ethics*. This article does not report research on animal or human subjects, and ethics approval was therefore not required. The objects of measurement are software artefacts: authored fixtures, scaffold code paths, and the scaffold’s own emissions. The batteries are operator-authored prompts, not user data; battery runs are executed against fixtures, never against learner conversations. The one instrument that touches live traffic—the observe-only production drift detector of §4.8—matches patterns against platform-generated replies, alters nothing, and writes rows that are service-role only under deny-by-default row-level security, never surfaced to learners; no content from those rows, and no learner-authored content of any kind, is reproduced in this article. The learner-facing memory surfaces named in §3 are described architecturally, and no data drawn from them are reported.

### 4.11 Independent verification of one battery

The empirical honesty frame of §1.4 relies on a single admission: the batteries were authored by the operator, and the classification of replies against screens is therefore not independent of the party whose scaffold is under test. The deterministic design of the L1 screens (§4.3) is what makes that admission recoverable—a screen verdict is a mechanical fact about a reply, reproducible by anyone holding the reply and the screen definition—and this subsection reports the first exercise of that recoverability by a party outside the author.

One assessor, Matt Wilmot, an independent consultant with no part in authoring the fixtures, the screens, or the recorded verdicts, re-scored the nexus-reasoning battery working solely from the released fixtures kit (§4.10): the 10 prompts, the two screen definitions the battery names (hedging and telemetry), and the 10 recorded replies in the human-readable rendering the kit provides. The assessor scored every reply against every pattern before consulting the recorded verdicts and confirmed having worked only from the public repository. The exercise poses a narrow, checkable question—do the paper’s recorded verdicts for this battery reproduce when an outsider applies the released screens to the released replies?—and its answer is the substance of the verification.

The verdicts agreed in full. The assessor recorded zero hedging hits across the 10 replies, a single telemetry hit on reply 5, a monotony of 0.5 (five of 10 replies classified enumerated), and an overall verdict of fail at L1—because the pass rule requires both zero hits and monotony at or below the 0.6 threshold, and the one telemetry hit fails the first condition, though the monotony condition is met. Every figure matches the recorded run of 10 September 2026 exactly, including the identity of the single hit and the per-reply scaffold classification. Within the narrow scope of one battery, the recorded verdicts are what the released materials mechanically produce in an outsider’s hands.

The assessment also produced a finding that the harness records rather than suppresses, and it concerns the one hit. The telemetry screen matches a literal percent sign, on the reasoning that a percent sign in learner-facing text is characteristically a telemetry leak—a confidence figure or a detection rate escaping into prose the observation lock exists to keep clean. The hit on reply 5 is a percent sign used differently: as an algebraic placeholder, “M%,” naming a hypothetical threshold inside a reasoning move on the Nexus surface, not a measurement reported to a learner. The assessor flagged this as a case where the mechanical verdict and the screen’s intent diverge—the pattern fired correctly, but on a use the screen was not built to catch. This is a screen false positive, and it is reported here as a result of the verification rather than corrected away, because it is exactly the kind of boundary case that an operator scoring alone is least likely to notice and an independent scorer is most likely to identify. It bears directly on H2 and on the telemetry screen’s role in the observation-lock predictions: a screen that over-matches is a screen whose precision must itself be characterised before its verdicts carry enforcement weight, which is the same discipline the observe-only detector applies to its own patterns (§4.8) and which the deferred L2 layer (§4.4) exists to supply. The finding narrows rather than widens the article’s claims: it confirms that the L1 verdict is reproducible while showing that reproducibility and appropriateness are distinct properties, the second of which requires an independent eye to judge.

A second assessor subsequently re-scored the same battery from the same released kit: Savvas Savva, a doctoral researcher at the School of Engineering, Frederick University—the platform’s research partner for the follow-on learner-outcome study, an affiliation disclosed here in the same sentence as his result—who had no part in designing or authoring the battery, the fixtures, the screens, or the recorded verdicts. His verdicts agree in full with the recorded run and with the first assessor’s, on every measure: zero hedging hits across the 10 replies, the single telemetry hit on reply 5, a monotony of 0.5, and a fail at L1. Because of the partner affiliation, this subsection’s independence claim continues to rest on the first assessor alone; the second re-scoring is reported as additional reproduction evidence—two assessors, one of them wholly outside the programme, in complete agreement on every figure including the identity of the single hit—with its provenance stated. His verdict set, declaration, and the affiliation disclosure are published in the fixtures kit repository (§4.10). A third assessor, Simon Alsop, Managing Director and founder of Learning Skills Partnership Ltd, re-scored the same battery from the released kit. His scores, recorded before consulting the published verdicts, agreed fully with the recorded run and both previous assessments. This is additional reproduction evidence; the assessor is not independent of the operator.

The verification is one battery scored by three assessors outside the author—the independence claim resting on the first—and it is reported as exactly that: not as an independent replication of the platform’s behaviour, which remains future work (§7.3), but as confirmation that the deterministic layer’s verdicts survive contact with scorers who had no hand in authoring them, together with one substantive finding that only such a scorer was positioned to make. The assessors’ verdict sets and declarations are published in the fixtures kit repository (§4.10) so that the verification is itself checkable rather than merely asserted.

## 5 The interaction side: explaining relevance

A paper focused on intelligent technology owes its readers a discussion of the other side—the interaction of users with the system and the likely impact of the advance on that interaction. This section provides it as a first-order argument rather than an appended courtesy. The argument is that three interaction properties—an observation lock, provenance grounding, and a two-register voice law—are what the harness measures conformance to, and that these properties become testable precisely because they are structurally enforced. The interaction side is therefore not separable from the technology side: the harness is an instrument for holding an interaction contract, and the interaction contract is what makes the harness worth building.

### 5.1 The observation lock as user-facing law

The observation lock governs what a user experiences when an interactive intelligent system reflects their conversation back to them. The scaffold surfaces a Current Reflection panel—the conversation-shape table’s structured self-description, described in the scaffold section—and the lock states its constraint plainly: the panel describes the conversation, never the person. A user reading the panel encounters a description of the exchange as a shared object between them and the system—its centre of gravity, its live tension, its carried question—not a verdict rendered on the user as a subject to be characterised.

The distinction is consequential for the experience of being observed by an AI. A system that tells a user what the system has concluded about them positions the user as an object under assessment; a system that reflects the shape of the conversation positions the user as a participant in a shared act of noticing. The lock makes this a matter of enforcement rather than intention: second-person address and telemetry vocabulary are stripped from surfaced fields by a validator, not left to the substrate’s discretion. The learner-as-subject and telemetry-vocabulary screens described in the methodology are the measurement-time counterparts of this serving-time enforcement: they exist to catch, in a regression run, any drift back towards the framing the lock forbids. The user’s experience of not being characterised is, in this design, a property with an instrument pointed at it.

### 5.2 Provenance grounding as user-facing law

Provenance grounding governs what a user can learn when they interrogate the system about itself. Every authenticated Nexus turn carries a factual WHAT YOU CAN SEE block, and the system is bound to answer architecture questions from that block alone—revealing provenance rather than reasoning, and, where a state read fails, stating “unknown this turn” rather than improvising. A user who asks the system what it can see about them receives an answer grounded in the same code paths that gate the real capabilities, not a plausible-sounding account the system has generated to satisfy the question.

The change to the interaction is a form of intelligibility. Ordinarily, a user interrogating a language-model-based system about its own state is at the mercy of the system’s fluency: the system will produce a confident, coherent self-description whether or not that description is true, and the user has no way to tell the two apart. Provenance grounding gives the user a system whose self-description is bounded by what it can verify and whose failures are declared rather than concealed. Soft-fail honesty—the rendering of a failed read as “unknown this turn”—is the interaction-level expression of a design commitment to declared ignorance over confident confabulation. The nexus-boundary battery and the reflection-move and subject-framing screens are the instruments that uphold this contract, testing whether the system, under direct interrogation, asserts state that its block does not contain.

### 5.3 The voice law as interaction law

The two-register voice law is itself an interaction contract. The Nexus register offers long-form, structured reasoning with competing hypotheses and disconfirmers because a user of that surface is trying to understand a system and needs the material to weigh. The Clara register offers one observation, one question, then stops, because a user of that surface is trying to notice something and a long reply would do the noticing for them. The brevity law is not a stylistic preference but a positional intervention with measurable interaction consequences: a terse reply that ends on a question hands the next move back to the user, whereas a long reply keeps it with the system.

This is why the brevity law is stated as one of the article’s predictions and enforced twice—at the system-prompt level and again through a tail-position demand. The interaction consequence the law is meant to produce—the user, not the system, doing the reflective work—is measurable through the mean reply length and scaffold-monotony metrics, and the prediction is precisely that the intervention cuts words while preserving structural variety and constraint discipline—the operationalisation §6.2 (H3) states in full. The voice law thus connects an interaction commitment to a measurable quantity, which is the pattern the whole interaction section instantiates.

### 5.4 Why this section is load-bearing, not decorative

The three properties above are not illustrations chosen to satisfy a reviewer’s expectation that interaction be discussed. They are the very things that the harness measures conformance to. The telemetry vocabulary and learner-as-subject screens exist because the observation lock is a real interaction contract that can be violated; the reflection move and subject framing screens exist because provenance grounding is a real interaction contract that can be violated; the length and monotony metrics exist because the voice law is a real interaction contract that can be violated. Remove the interaction contracts, and the screens have nothing to measure; remove the screens, and the interaction contracts have no instrument holding them.

The article therefore treats the interaction side not as a courtesy requirement but as a substantive one. An interactive intelligent system’s most consequential properties are frequently properties of the interaction—whether a user is characterised or accompanied, whether a user can trust the system’s account of itself, whether the system does the user’s reflective work or returns it to them. These properties are exactly the ones that a substrate-directed evaluation cannot see and that a scaffold-directed harness is built to hold. The interaction side is where the harness earns its keep, and discussing it explicitly is, on this reading, what obliges the paper to name what its instruments are ultimately for.

## 6 Eight falsifiable harness predictions

The empirical honesty caveat, stated here as the third of three placements. The eight predictions below are predictions, not settled findings, and they are offered for adversarial replication. The batteries that generate the evidence were authored by the platform’s operator; the classification of replies against screens is not independent of the party whose scaffold is under test; and the number of platforms studied is one. Each prediction is stated with four parts—the claim, the evidence observed so far on this platform, the runtime carrier that would let a reviewer check or contest it, and the falsification condition that states precisely what would count against the claim. The evidence is reported as observed on this platform at the primary anchor; it supports each claim as an observation, not as a demonstration, and the falsification conditions are the article’s invitation to test the claims against something other than the platform’s own data.

### 6.1 Relationship to the companion paper’s pre-registered outcome predictions

The companion paper pre-registers eight outcome predictions of its own, F1 through F8, concerning the deployed system’s behaviour—constitutional-clause referencing incidence (F1), substrate-call latency-budget adherence (F2), the recall, precision, and F1 score of the prescriptive-language detector measured against a hand-labelled corpus (F3), the constitutional runtime’s effect on prescriptive-language incidence under ablation (F4), Portrait-referencing incidence and consistency (F5), decision-continuity when the Portrait is populated (F6), reflection-cycle completion and its effect on next-move conformance (F7), and reflection-driven Portrait revision (F8) (Dobson, 2026b). These are outcome predictions: claims about what the deployed system’s behaviour will demonstrate when measured. The eight predictions of this article, numbered H1 through H8 to keep the two sets unambiguous, are harness predictions: claims about the measurement instruments themselves—that a positional variable dominates emission, that an enforcement point prevents a class of generation, and that a drift is detected within a bounded window.

The two sets converge through the instruments. The turn ledger’s per-turn latency field is the instrument for F2; the observe-only detector of §4.8, along with the quarantine, accumulates the hand-labelled corpus F3 requires, and F3’s measurement is in turn the gate on any enforcement extension of that detector; the ablation switch F4 requires is measurement apparatus scoped to the outcome study and does not yet exist, which is stated here so the commitment is visible; and the noticings, Portrait, and conversation-shape instruments of §3.2, read through the health monitor, are the carriers for F5 through F8. The numerical floors and thresholds for F1 through F8 are to be registered against the baselines the health monitor establishes—the same fortnight-baseline discipline that governs the harness’s own bands—and registering them before the outcome study begins is a stated obligation of that study, not a step this article claims to have completed. What this article contributes to the outcome programme is therefore exactly what a harness paper should: the instruments, their own falsifiable characterisation, and an honest statement of which apparatus is still owed. For completeness, a third numbered set exists in the programme and is deliberately not restated here: the foundation paper states four theory-level architectural predictions of its own (Dobson, 2026a)—comparative claims requiring probing across matched architectures—which neither the companion’s outcome predictions nor this article’s harness predictions test or depend on. The three sets are disjoint by construction: the foundation paper’s predictions would falsify the theory, F1 through F8 would falsify claims about the deployed system’s behaviour, and H1 through H8 would falsify claims about the instruments that measure it.

### 6.2 Observed on this platform (H1, H2, H3, H5, H7)

The five predictions in this subsection carry dated observations from the platform itself, under the operator-authored frame of §1.4. These are observations rather than demonstrations: the batteries were authored by the platform’s operator, the classification of outputs against screens is not independent of the party whose scaffold is under test, and no observation reported here has been independently replicated. A reader who reaches this subsection without having read §1.4 should approach the five predictions below with that understanding. Each is stated with four parts: the claim, the evidence observed so far, the runtime carrier that would allow a reviewer to check or contest it, and the falsification condition.

#### 6.2.1 H1—the positional law

*The claim*. Instrument-emission compliance under transcript-plus-instructions prompting is dominated by the position of the instruction: a one-line demand placed at the end of the transcript produces emission, whereas an identical mid-prompt contract produces near-silence, on the same substrate and with the same content.

*Evidence so far*. On the platform, the shape instrument emitted at or near zero under a mid-prompt contract and emitted reliably after a tail demand was added on the same day, with no other change; the noticings channel showed the same reversal. The change was observed against the deploys of 16 July 2026. The finding is worth stating carefully because it runs counter to natural intuition. A designer who has written a clear, complete instruction contract into the middle of a prompt would expect the substrate to honour it, and the observed near-silence is initially surprising: nothing was wrong with the contract’s content, only with its position relative to the transcript that followed it. The intervention that restored emission was minimal — a single line of demand relocated to the end—and its effect was not marginal but categorical, shifting from near-zero emission to reliable emission. This is precisely the kind of scaffold regression that the introduction argued is hard to detect: the substrate was functioning, the contract was present, and yet a named epistemic commitment was silently not being met.

*Carrier*. The field clara_turn_ledger.lock_checks.shape records, per turn, whether the shape instrument’s emission cleared its check, and the emission rate before and after the positional change is read directly from it. Because the ledger holds one row per assistant turn per surface, the before-and-after comparison is a query against a single table rather than a reconstruction from logs, which is what makes the claim checkable rather than merely asserted.

*Falsification*. An A/B comparison across at least 200 turns, with instruction position as the only variable, showing no material difference in emission rate, would falsify the claim. The falsification condition is deliberately demanding of the claim rather than of its critic: it fixes the substrate, the content, and every other variable, isolates position, and requires a sample large enough that a null result cannot be attributed to noise. A single well-run A/B of this kind, on this platform or another, would settle the matter, and the article invites it.

#### 6.2.2 H2—composition-time assertion prevents ungoverned generation on the path it gates

*The claim*. Fail-closed checks applied at composition time yield zero learner-visible governed-pattern violations on the composition-gated path, at near-zero marginal cost, because a rejected move is never generated in the first place.

*Evidence so far*. The ordering of assertConstitutional before generation is inspectable in code, and the residual-strip and quarantine counts show zero learner-visible governed-pattern violations on the composition-gated path at the primary anchor. The near-zero marginal cost follows from the enforcement point: a rejected move never incurs the substrate round-trip that dominates a turn’s cost. The scope of the claim must be stated with the same care as the claim itself. The composition-time layer gates candidate moves composed by the scaffold; free-text replies generated directly by the substrate on conversational surfaces take a different path, and drift on that path is the subject of the observe-only detector of §4.8 and the companion paper’s outcome predictions F3 and F4, not of this prediction. H2 is not a claim that the pattern list is complete, nor that every surface is composition-gated; it asserts that whatever the composition-time layer gates is gated before generation rather than after it, so that on that path the substrate is never given the opportunity to emit the governed pattern at all. This restates, in directly testable form, the enforcement-point distinction that the companion paper argues conceptually (Dobson, 2026b).

*Carrier*. The ordering of assertConstitutional is inspectable in the code path directly and is validated by a dedicated wiring test that fails the build if the composite ceases to run on the compose path; the residual-strip counts and the arrival counts in clara_emission_quarantine provide the audit trail for any block that escaped composition-time rejection. The carriers work together: the code-path ordering and its test establish that the check runs before generation, and the counts determine whether anything the check should have caught nonetheless reached a learner.

*Falsification*. Any learner-visible output embodying a pattern that the composition-time layer claims to gate, generated from a move that passed the check, would falsify the claim directly. A violation reaching a learner through a route that never passed the check is a routing gap rather than a falsification of the enforcement-point claim—and the honest accounting of exactly such routes is why the observe-only detector exists and why the companion paper’s F4 pre-registers an ablation test rather than assuming the answer.

#### 6.2.3 H3—the brevity intervention

*The claim*. An editorial law enforced at both the system-prompt level and the transcript tail reduces mean reply length on the Clara surface by 30 to 40 per cent and holds scaffold monotony below 0.6, while keeping the L1 constraint screens at zero hits—words are cut while structural variety (the monotony metric) and constraint discipline (the L1 screens) are preserved.

*Evidence so far*. The observed basis is the 17 July baseline: the hand-scored operator transcript that recorded a monotony of 0.8—eight of 10 replies carrying the same numbered scaffold—and the reply lengths against which the 18 July brevity law set its 30–40% target. The instrumented carrier is built and is the row this claim is read from: clara_eval_runs records meanReplyChars, monotony, and screen hits together per run, dual-stamped with git SHA and model label. The first fully persisted baseline runs against that carrier were recorded on 10 September 2026, during this article’s revision—six batteries, each row dual-stamped—so the instrumented path now carries live rows; the reduction magnitude against the hand-scored baseline is a reading that the instrumented series carries forward from those rows, and the distinction between the hand-scored basis and the instrumented series is stated here rather than smoothed over. The claim’s three quantities—length reduction, monotony, and screen hits—are recorded together per run so that a reduction achieved at the cost of a screen regression would be visible rather than hidden. The joint recording is the methodologically important part. A brevity intervention that simply truncated replies could achieve a large length reduction while degrading the reply into a monotonous, screen-failing stub, and a claim that reported only the length reduction would conceal exactly that degradation. By binding the three quantities into a single run, the harness makes the claim’s most likely failure mode visible: the prediction is not merely that words are cut, but that they are cut without the reply collapsing into repetitive form or losing its constraint discipline, and all three conditions are read from the same row. The word depth would overstate what is measured, and this claim deliberately avoids it: what H3 operationalises as preserved is exactly the conjunction of these two measured properties—structural variety above the monotony floor and zero regressions on the constraint screens. Whether the shorter replies preserve depth in any richer sense is a judgement that the deterministic screens cannot render; the instrument for it would be the L2 rubric layer, and that layer is deferred (§4.4).

*Carrier*. The table clara_eval_runs, specifically the fields meanReplyChars and monotony, and the recorded screen hits, measured across git SHAs, with the 17 July transcript as the baseline. The baseline is a specific, dated run rather than a notional prior state, which means the comparison is between two measured points and not between a measured point and a remembered impression.

*Falsification*. A post-law battery run showing a length reduction below 15 per cent, or a monotony above the 0.6 threshold, or any regression on the constraint screens, would falsify the claim. The three-part falsification condition mirrors the three-part claim: any one of the three failing is sufficient to falsify, which is a stronger and more easily falsified position than a claim that would survive on the strength of the length reduction alone.

#### 6.2.4 H5—provenance grounding eliminates self-description confabulation

*The claim*. A per-turn factual capability block reduces unverifiable account-state assertions on the boundary battery to zero, in a setting where the ungrounded system produced them reliably.

*Evidence so far*. The ungrounded system, in the 18 July transcript, produced three improvised state claims, one contradiction requiring repair, and one learner-as-subject framing; the grounded system, run against the boundary battery at the primary anchor, produced none of these on the platform. The prediction is that grounding, once run repeatedly against the fixture, continues to hold the system to its block. The before-and-after contrast is the substance of the claim. Confabulated self-description is a characteristic failure of a fluent substrate asked about its own state: it produces a plausible, coherent account whether or not the account is true, and the three improvised state claims on the ungrounded transcript are instances of exactly this. Grounding does not ask the substrate to be more truthful; it removes the substrate’s licence to answer from anything other than the factual block, so that a claim the block does not support cannot be made in the first place. The prediction is that this structural constraint, not an appeal to the substrate’s good behaviour, is what drives the improvised claims to zero.

*Carrier*. The nexus-boundary-v1 fixture; the subject-framing and reflection-move screens; and the L2 rubric keys authored for the boundary prompts. The fixture supplies the adversarial prompts; the two screens catch the two named defects deterministically; the rubric keys are the L2 layer that adds a graded judgement of self-description honesty beyond the deterministic screens (first exercised at revision time, §4.4; unexercised at both anchors)—the one place among the eight predictions where the deferred L2 layer is explicitly named as part of the carrier, and correspondingly the place where the harness’s own incompleteness is most visible.

*Falsification*. A grounded run that still asserts state the WHAT YOU CAN SEE block does not contain, or that contradicts the block, would falsify the claim. The two disjuncts cover the two ways grounding could fail: asserting state absent from the block (fabrication) and contradicting state present in the block (inconsistency). A grounded run exhibiting either would show that the structural constraint did not, in fact, bind the substrate’s self-description, which is the whole of what H5 claims.

#### 6.2.5 H7—the observation lock is achievable live

*The claim*. A turn-cadenced, learner-visible reflection of the conversation can run in production with zero leaked structural blocks and zero second-person or telemetry vocabulary in surfaced fields, under real load.

*Evidence so far*. The unconditional strip is pinned by test; the validator field drops, quarantine counts, and residual-strip counts have all read zero leaks on the platform since the 17 July fix. The claim is that this zero-leak state is achievable and sustainable live, not merely under test conditions. The distinction between achievable-under-test and achievable-live matters because a strip that passes a unit test can still fail in production if an unexpected block shape escapes it under real traffic. The claim is stated at the level of the harder condition—under real load—precisely because the observation lock is an interaction contract, and an interaction contract that holds only in the test harness is not held at all from the user’s point of view.

*Carrier*. The unconditional strip, pinned by a test that fails the build on regression; the validator field drops; the quarantine counts; and the residual-strip counts. The four carriers form a layered defence: the unconditional strip removes structural blocks before validation, the validator drops any prohibited field that survives, the residual strip catches anything that escaped both, and the quarantine retains whatever was caught so that a leak attempt leaves a trace even when it is successfully blocked. A reviewer checking H7 reads all four—the test to confirm the strip is enforced and the three counts to confirm nothing surfaced.

*Falsification*. Any surfaced structural block or any validated field found to contain a prohibited class—second-person address or telemetry vocabulary—would falsify the claim. The falsification condition is a single observed leak, which makes H7 among the most exposed of the eight predictions: it does not claim a low leak rate but a zero leak rate, and a single counter-instance suffices to falsify it. This is a deliberate choice because an observation lock that leaks occasionally does not deliver the interaction property it promises—a user cannot rely on not being characterised if the lock holds only most of the time.

### 6.3 Prospective (H4, H6, H8)

The three predictions in this subsection concern experiments that have not yet occurred—the vendor-change test (H4), the seeded-drift test (H6), and the post-fix accumulation window (H8). They are stated with the same four parts, but their evidence paragraphs describe the apparatus in place and the reading that would count, not results; nothing in this subsection is offered as observed.

#### 6.3.1 H4—compliance is scaffold-borne, not substrate-borne

*The claim*. Constraint-holding under adversarial prohibition—the clara-constraint battery’s zero-jargon, zero-advice result—survives substrate model updates unchanged because the discipline lives in the scaffold rather than the model.

*Evidence so far*. Battery runs are stamped with both model_label and git_sha, making the two factors separable by construction; on the platform, the constraint result held across the model labels recorded at the primary anchor. The prediction is that this separability, once exercised across a genuine vendor model change, will show the scaffold factor carrying the compliance. This prediction is the empirical counterpart of the whole scaffold-versus-substrate distinction that organises the companion paper (Dobson, 2026b). If compliance were substrate-borne, a vendor model update would move the screen results even with the scaffold unchanged; if it is scaffold-borne, the results would hold across the update. The design that makes this testable is the dual stamp: because every run records both which scaffold and which model produced it, a later analyst can hold one coordinate fixed and vary the other, which is exactly the manipulation the claim requires. The prediction is honest about its current evidentiary limit—a genuine vendor model change large enough to constitute a real test had not been exercised at the primary anchor—so the claim rests on the separability being built in, awaiting the natural experiment that a vendor update will eventually supply. The platform’s substrate policy makes that experiment routine rather than exceptional: substrates are seat-scoped, no substrate changes silently, and every promotion is battery-gated against pre-registered acceptance deltas, so each vendor promotion arrives with exactly the paired runs H4 requires.

*Carrier*. Battery runs in clara_eval_runs are stamped with model_label and git_sha—the two coordinates that allow a compliance result to be attributed to the scaffold or the substrate rather than being confounded between them. Without both stamps, the attribution would be impossible; with both, it is a matter of grouping runs by one coordinate while the other varies.

*Falsification*. A vendor model update that materially changes the L1 screen results with the scaffold held constant—that is, with git_sha fixed and only model_label changing—would falsify the claim. The condition is symmetric and fair: it does not ask that the scaffold be perfect, only that the compliance does not evaporate when the model beneath it changes, which is precisely what the scaffold-borne hypothesis predicts.

#### 6.3.2 H6—bounded drift detection

*The claim*. The quarantine-and-health-monitor pair bounds the detection latency of an emission-contract drift to at most 14 days, against an observed 60-plus days for the same defect class before the instruments existed.

*Evidence so far*. The pre-instrument defect class went undetected for 60-plus days on the platform; the instruments, once their bands are armed, sweep weekly, which bounds the detection latency to at most two sweeps. This claim is the one most conditioned by the honest empirical frame, because the bands for the first cohort of instruments armed only on 20 July 2026, and the first fortnight of data is baseline-establishing rather than detection-capable—the detection bound is a prediction about the instruments’ behaviour once armed, not an observation of a detection that has already occurred.

*Carrier*. The arrivals in clara_emission_quarantine; the weekly rows in clara_instrument_health; and the handoff queue into which an out-of-band rate raises a work item. The three carriers form a chain: a drifting emitter deposits malformed blocks into the quarantine, the weekly health sweep interprets the rising arrival rate as out of band, and the handoff queue conveys the finding to a human. The detection latency that the claim bounds is the length of that chain, and its upper bound of 14 days follows directly from the sweep being weekly—at most two sweeps must pass before an out-of-band rate is observed.

*Falsification*. A seeded synthetic drift—a deliberately malformed emitter placed behind a flag—surviving two weekly sweeps undetected would falsify the claim. This is the article’s clearest example of a falsification condition that can be executed deliberately rather than awaited: the operator, or an adversarial replicator, can inject a known drift behind a flag and observe whether the instruments detect it within the claimed window. A drift that survives two sweeps would demonstrate that the detection bound is false, and the deliberateness of the test is a strength, because it does not depend on a natural drift occurring during an observation period.

#### 6.3.3 H8—the developmental substrate accumulates at honest rates

*The claim*. With the positional fix and the emission discipline in place, the noticings channel accumulates typed developmental observations at a stable per-active-learner rate sufficient for the longitudinal instruments, materially above the pre-fix platform-wide baseline of approximately four per fortnight.

*Evidence so far*. The pre-fix rate was approximately four noticings per fortnight platform-wide; the positional fix and emission discipline are expected to raise the rate materially, and the bands against which the post-fix rate is judged are to be set from the first fortnight’s baseline, established on 20 July 2026. As with H6, the honest reading is that this is a prediction about a rate whose measurement window has only just opened.

*Carrier*. The clara_nexus_noticings counts recorded in clara_instrument_health, with bands set from the first fortnight’s baseline. This prediction closes the loop with H1: the positional law’s near-zero emission was, in effect, starving the developmental substrate, and the pre-fix rate of approximately four noticings per fortnight platform-wide is the downstream signature of that starvation. H8 predicts that fixing the positional cause raises the downstream effect, and the carrier is the same health-monitor table that holds the other rate-based predictions, read for the specific quantity of noticings accumulation.

*Falsification*. Post-fix rates that are statistically indistinguishable from the pre-fix baseline over a month of sweeps would falsify the claim. The word “materially” in the claim, and “statistically indistinguishable” in the falsification, are load-bearing and honest: H8 does not predict a specific multiple, only a material rise above a very low baseline, and it accepts falsification if the rise cannot be distinguished from noise over a month. This is the least precise of the eight predictions, and the article does not pretend otherwise; its evidentiary status is further weakened by the bands having been armed only on 20 July 2026, so that the month of sweeps required to test it had not elapsed at the primary anchor.

## 7 Discussion

### 7.1 The priority claim, stated narrowly

The article makes a priority claim, and it is stated as narrowly as the source material permits, because a broad claim would be both false and easily falsifiable. The individual ingredients have prior art that the related-work section cites: evaluation harnesses and regression testing of prompts are established practices, guardrail filters are commonplace, and design-by-contract has been around for decades. The claim that survives scrutiny concerns a combination, not any single ingredient.

To the author’s knowledge, no prior published system documents the combination of, first, fail-closed constitutional assertion at prompt-composition time; second, a learner-facing live reflection instrument governed by an enforced observation lock; third, per-turn factual provenance grounding derived from the capability-gating code paths themselves; and fourth, an integrated self-measurement layer in which operator-authored adversarial batteries, deterministic defect screens, emission quarantine, instrument-health monitoring, and an observe-only production drift detector gate and audit the same production scaffold they measure. The claim is offered in the falsifiable form that suits it: a single documented counter-example predating this report would falsify it, and the article invites exactly that. The priority being claimed is for the combination as an integrated whole running in production, not for any of its parts, and the narrowness is deliberate.

### 7.2 What the harness does not yet do

Four honest limits mark the harness’s current perimeter, and each is stated plainly rather than concealed. Two are inherited from the companion paper and are not closed by this article. The compute-budget controller the companion paper reports as a partial realisation—a signal captured per selector decision but read by no reflective controller—remains unbuilt, and the health monitor and batteries do nothing to change that (Dobson, 2026b). The runtime conflict detector the companion paper names as a v-next carrier for the Perceptual Control Theory layer remains unbuilt for the same reasons the companion paper gives (Dobson, 2026b).

The third limit is shared by both papers and is stated identically in both: the matched-cohort clinical protocol—cohort matching criteria, a primary outcome, a stop rule—remains unauthored. This article delivers the instruments and the companion paper delivers the pre-registered outcome predictions, but the protocol that would turn a subset of those predictions into a matched-cohort test with a stop rule is a distinct piece of research design that neither paper claims, and registering the F-set’s numerical floors ahead of the outcome study is part of that still-owed work (§6.1).

The fourth limit is opened by this article rather than inherited. The L2 model-judged scoring layer was authored—rubric keys exist, versioned per prompt—but was unexercised throughout the entire measurement period, deferred by the design argument stated in the methodology; its first exercise came only at revision time (§4.4) and contributes no evidence to any prediction here. The omission for the reported period is genuine and is identified as such: the article does not present L1’s deterministic verdicts as a substitute for a judgement that L2 would supply.

### 7.3 The methodology contribution as a reusable pattern

Abstracted from the specific tables and code paths that realise it here, the harness is a five-part pattern for measuring any interactive scaffold that sits over an opaque substrate. The five parts are fixtures, screens, quarantine, health monitoring, and production observation. Fixtures are adversarial batteries authored to encode named defects rather than to sample representative use or reach a coverage bar. Screens are deterministic invariants that fail closed on those defects, providing mechanical verdicts that do not share their subject’s failure mode. Quarantine treats failure evidence as a first-class object, converting a discarded failure—a lost signal—into a durable, queryable measurement. Health monitoring acts as an autonomous handoff channel, placing the burden of vigilance on the system rather than on a human’s attention to a dashboard. Production observation runs the pattern screens against live traffic without altering it, accumulating the labelled corpus on which any future enforcement extension must earn its authority.

The pattern’s generality follows from the generality of the problem it addresses. Any builder surrounding an unretrainable substrate with a scaffold that enforces a named epistemology faces the same difficulty this article opened with: the scaffold’s own regressions are hard to catch because the instruments in common use point at the substrate. The five-part pattern directs an instrument at the scaffold, and it does so with components that make no assumptions about the particular epistemology being enforced—only that the epistemology can be stated as named defects a screen can detect. This is what the article offers beyond the single platform it reports.

### 7.4 Extension to learning loops: measure before you adapt

A scaffold that enforces is one measurement problem; a scaffold that adapts is a harder one, because an adapting scaffold is a moving target whose drift can no longer be distinguished from its learning by inspection alone. The platform’s first scaffold-level learning loop was shipped, at the addendum anchor, under the discipline this article describes, and the case is reported because it generalises.

The loop is a Bayesian thinker-weights posterior-one Beta prior per practitioner per developmental instrument, held at a uniform prior until evidence arrives—whose write path updates only from typed feedback events (a completed cycle feedback reinforces the instruments that served that cycle) and whose read path orders instrument selection by posterior mean, with the previous deterministic order as a tie-break. Three properties of the shipment express the measurement discipline. First, a test-pinned uniform-posterior guarantee: with an unpopulated posterior, selection is byte-identical to the pre-learning scaffold, so the loop’s introduction changed nothing on day one and can change behaviour only as evidence accumulates. Second, typed evidence only: the update rule reads structured events, never free text, so the learning signal inherits the same auditability as the constitutional layer’s typed checks. Third, ledgered observability: the posterior lives in a dedicated table whose row counts, priors, and update timestamps are readable by the same health-monitoring cadence that watches the other instruments, so the loop’s activity is itself a monitored rate.

The generalisation is the point. A learning loop introduced without these properties is an unaudited drift source indistinguishable from the regressions this article’s instruments exist to catch; a learning loop introduced with them is a measured variable—its no-op baseline pinned by test, its evidence typed, its activity on the same dashboards that page as everything else. The pattern’s extension to adaptation is therefore one sentence long: a scaffold may learn only through carriers the harness can read, and every learned change must be behaviourally silent until evidence, not deployment, activates it. The same rule will govern the loop’s planned extensions—attenuation from typed abandonment events, and estimator population for the constitutional layer’s declared annotations—each of which enters as a measured variable or does not enter at all.

### 7.5 Limitations

The empirical honesty caveat is reprised once more, deliberately, as the frame for every quantitative statement in the article: the predictions are predictions, the batteries were operator-authored, the classification is not independent, and the number of platforms studied is one. Several specific limitations follow from this frame. Generalisation from a single platform is unwarranted without replication; the article’s contribution is a pattern and a set of falsifiable predictions, not a demonstrated general result. The operator-author caveat means the party that authored the adversarial batteries is the party whose scaffold they test, so the batteries may under-probe defects that the operator did not anticipate. The classification of replies against screens is likewise non-independent, and although the deterministic screens reduce this concern by making verdicts mechanical, the choice of which defects to screen for remains the operator’s. The health monitor’s bands arm only after a fourteen-day baseline. The baseline period is not detection-capable; for the first cohort, the bands armed on 20 July 2026. This directly conditions the drift-detection and accumulation-rate predictions. Finally, the observe-only detector reported at the addendum anchor had, at the time of writing, only begun accumulating its corpus; its error characteristics are the subject of the companion paper’s outcome prediction F3 and are explicitly unmeasured here.

### 7.6 A note on explaining relevance

A reader who approaches this article primarily interested in interaction, rather than in the measurement technology, should take a specific claim from it. The interaction properties that matter most in an interactive intelligent system—whether a user is characterised or accompanied, whether a user can trust the system’s account of itself, whether the system does a user’s reflective work or returns it—are properties of the scaffold, not the substrate, and they are invisible to any evaluation directed at the model. Making them hold requires enforcing them structurally; knowing they hold requires an instrument pointed at the enforcement. The harness reported here is that instrument, and its predictions are, at bottom, predictions about whether an interaction contract can be held under load and whether its breach can be caught quickly. On this reading, requiring a technology paper to discuss the interaction side is not a formality but a discipline: it obliges the author to clarify what the technology is ultimately for, and for this technology, the answer is the interaction contract it exists to hold accountable. For a reader of this specialty section, the connection to human learning and behaviour change can be summarised in one sentence: every instrument in this harness measures a precondition of trustworthy learning measurement—that the learner is reflected and not characterised, that the system’s self-account can be believed, that the reflective work returns to the learner rather than being done for them, and that the developmental substrate accumulates at honest rates—and the learner-outcome study those preconditions serve carries pre-registered predictions and is downstream, not skipped.

### 8. Conclusion

The methodological contribution of this article is a self-measurement layer for an interactive scaffold over an opaque substrate, reported as built and abstracted as a reusable five-part pattern: adversarial fixtures that encode named defects, deterministic screens that fail closed on them, quarantine that treats failure evidence as a first-class object, health monitoring that hands off autonomously, and production observation that accumulates labelled evidence without altering behaviour. The layer’s six components each carry a named runtime carrier, and the pattern’s premise is the one the introduction opened with: in systems built by surrounding a model, quality regressions are frequently scaffold-borne, and catching them requires instruments pointed at the scaffold.

The evidentiary status of the harness is stated once more because it frames everything the article claims. The batteries were authored by the platform’s operator; the classification of replies against screens is not independent of the party under test; and the number of platforms studied is one. Eight harness predictions are stated, each with a named carrier and an explicit falsification condition, and the article’s structure now carries their evidentiary split: five (H1, H2, H3, H5, H7) rest on dated on-platform observations and are reported in §6.2; three (H4, H6, H8) are prospective and are reported under that label in §6.3 (Table 1 carries the same distinction row by row and Table 2 compares this harness with adjacent approaches). An observed status is an observation under this frame, never a demonstration, and no prediction has been independently replicated. The harness’s model-judged L2 layer, deferred throughout the measurement period this article reports, was first exercised during revision (10 September 2026): a judge model from a different provider than the substrate returned rubric verdicts across all six fixtures in the same runs. Those verdicts are recorded with the runs and released with the fixtures kit; they are a first exercise, not a validated instrument, and the L2 layer’s systematic use remains future work.

**Table 1:** H1–H8 summary.

ID	Claim	Carrier	Falsification	Evidence status at the anchors
H1	Instruction position dominates emission compliance	clara_turn_ledger.lock_checks.shape	A/B ≥ 200 turns with position as the sole variable showing no material difference	Observed: categorical near-zero-to-reliable reversal on the same day (16 July); the controlled A/B is prospective
H2	Composition-time assertion prevents ungoverned generation on the path it gates, at near-zero cost	assertConstitutional ordering (test-pinned); residual-strip and quarantine counts	Any learner-visible governed-pattern violation from a move that passed the check	Observed: ordering test-pinned; zero governed-pattern violations on the gated path at the primary anchor; adversarial counter-instance search prospective
H3	Brevity law reduces length 30–40%, monotony below 0.6, screens at zero	clara_eval_runs (meanReplyChars, monotony, screen hits); 17 July baseline monotony 0.8	Post-law runs showing <15% reduction, monotony above threshold, or any screen regression	Observed: 17 July hand-scored baseline (monotony 0.8); joint-recording instrument built; first persisted baseline runs recorded 10 September 2026—reduction magnitude read forward on the instrumented series
H4	Compliance is scaffold-borne, not substrate-borne	Battery runs stamped with model_label and git_sha	Vendor model update materially changing L1 results with scaffold constant	Prospective: separability built by the dual stamp; no vendor change large enough to test had occurred at the primary anchor
H5	Provenance grounding eliminates self-description confabulation	nexus-boundary-v1 fixture; subject_framing and reflection_move screens; L2 rubric keys	Grounded run asserting state absent from the block or contradicting state present in it	Observed: ungrounded 18 July transcript versus grounded battery run; repeated grounded runs prospective
H6	Drift detection latency bounded to ≤14 days	clara_emission_quarantine arrivals; clara_instrument_health weekly rows; handoff queue	Seeded synthetic drift surviving two weekly sweeps	Prospective: bands armed 20 July with no detection yet at the anchor; the seeded-drift test is runnable on demand
H7	Observation lock achievable live with zero leaks	Unconditional strip (test-pinned); validator field-drops; quarantine and residual-strip counts	Any surfaced structural block or validated field containing prohibited classes	Observed: zero leaks since the 17 July fix; the sustained live condition is the ongoing test
H8	Noticings accumulate above ~4/fortnight pre-fix baseline	clara_nexus_noticings counts in clara_instrument_health	Post-fix rate statistically indistinguishable from pre-fix over a month	Prospective: the measurement window opened 20 July; a month of sweeps is required

Each harness prediction is stated with its named runtime carrier, its falsification condition, and its evidence status at the anchors—distinguishing what has already been observed on this platform from what remains prospective. An “observed” status is an observation under the operator-authored frame of §1.4, never a demonstration, and no prediction has been independently replicated. The section structure follows this column: §6.2 carries the observed predictions, §6.3 the prospective.

**Table 2:** Enforcement-point comparison across five approaches to language-model system quality.

Dimension	This harness	Eval harness	Guardrail filter	Constitutional AI	Design-by-contract
Point of intervention	Measurement-time on fixtures, composition-time assertion, and observe-only production logging	Measurement-time against the substrate	Serving-time on model output	Training-time critique-and-revise	Defined execution boundary at a call site
Coverage target	Named defects drawn from observed scaffold drift	Broad capability or scenario coverage of the substrate	Configured rule set on outputs	Stated constitution of principles	Typed invariants at boundaries
Empirical frame	Falsifiable predictions with named carriers; operator-authored; n = 1; offered for replication	Benchmark scores; reproducibility-focused	Rule hit/miss at runtime	Preference-model and behavioural evaluation of the trained model	Correctness at contract boundaries
Failure-evidence policy	Quarantined as a first-class object with failure reason and 60-day purge; live matches logged unaltered	Recorded as benchmark results	Blocked or rewritten, typically logged	Revised output supersedes the original	Exception thrown; execution halts

The validation programme this article invites is therefore concrete. First, adversarial replication by independent operators, for which §4.10 states the minimum kit, is now released as a versioned open artefact—none of it requires this platform’s code, and a replication that fails to reproduce the predicted behaviours is exactly the result that the predictions are stated to invite. Second, the systematic running of the L2 rubric layer—first exercised at revision time—to supply, at scale and with stable keys, the graded judgements that the deterministic screens cannot render. Third, the registration of numerical floors for the companion paper’s outcome predictions against the baselines established by the health monitor, ahead of the outcome study those predictions define. Fourth, the matched-cohort clinical protocol that neither paper yet carries. The wider claim the pattern makes is one sentence long: a scaffold that enforces should be measured by instruments pointed at the scaffold, and a scaffold that adapts may learn only through carriers that those instruments can read.

## Data Availability

The Clara Nexus codebase is proprietary and owned by Astrala Advisory Services Ltd. The public fixtures kit is available at https://github.com/richarddobson1-cell/clara-harness-fixtures. Qualified reviewers may request inspection of the proprietary code and supporting records under a non-disclosure agreement against the two anchors in §1.5. Requests should be directed to the corresponding author. The companion paper’s external-audit protocol applies.

## Footnotes

1
https://github.com/richarddobson1-cell/clara-harness-fixtures
